# Influence of gelation on the retention of purple cactus pear extract in microencapsulated double emulsions

**DOI:** 10.1371/journal.pone.0227866

**Published:** 2020-01-16

**Authors:** Paz Robert, Cristina Vergara, Andrea Silva-Weiss, Fernando A. Osorio, Rocío Santander, Carmen Sáenz, Begoña Giménez

**Affiliations:** 1 Dpto. Ciencia de los Alimentos y Tecnología Química, Facultad de Ciencias Químicas y Farmacéuticas, Universidad de Chile, Santiago, Chile; 2 INIA La Platina, Instituto de Investigaciones Agropecuarias, Santiago, Chile; 3 Dpto. Ciencia y Tecnología de los Alimentos, Facultad Tecnológica, Universidad de Santiago de Chile, Santiago, Chile; 4 Dpto. de Ciencias del Ambiente, Facultad de Química y Biología, Universidad de Santiago de Chile, Santiago, Chile; 5 Dpto. de Agroindustria y Enología, Facultad de Ciencias Agronómicas, Universidad de Chile, Santiago, Chile; University of Queensland, AUSTRALIA

## Abstract

A purple cactus pear (*Opuntia ficus-indica*) extract (CP) was encapsulated in double emulsions (DE) gelled with gelatin (DE-CP-G) and with gelatin and transglutaminase (DE-CP-GT), as well as in a DE with a liquid external aqueous phase (DE-CP), in order to study the retention of betanin as colorant agent. Both gelled DEs showed a predominantly elastic behavior, in contrast with DE-CP. The degradation rate constant of betanin was significantly higher in DE-CP-GT (90.2 x 10^−3^ days^-1^) than in DE-CP-G (11.0 x 10^−3^ days^-1^) and DE-CP (14.6 x 10^−3^ days^-1^) during cold-storage (4 °C). A shift towards yellow color was found in all the systems during cold-storage (4 °C) and after thermal treatment (70°C/30 min), especially in DE-CP-GT, denoting a higher degradation of betanin. Betalamic acid, *cyclo*-Dopa 5-*O*-β-glucoside, 17-decarboxy-betanin and neobetanin were identified by UHPLC-MS/MS as degradation products of betanin.

## Introduction

Water-in-oil-in-water (W_1_/O/W_2_) double emulsions (DEs) are multi-compartmentalized systems in which droplets of a water-in-oil (W_1_/O) emulsion are dispersed within an outer continuous aqueous phase (W_2_) [1]. In recent years, there has been a growing interest in the incorporation of DEs in food applications, since they can be used as encapsulation and delivery systems for both hydrophilic and hydrophobic bioactive compounds due to their multi-compartmentalized structure. Furthermore, DEs have been proposed as a strategy for masking unpleasant flavors, developing reduced-fat and/or salt products and, by suitable formulation of the oil phase, improving the lipid profile [2].

Although DEs offer several advantages over simple oil-in-water emulsions, mainly the encapsulation, protection and release of both water-soluble and lipid-soluble compounds, as well as the reduction of the overall fat content of oil-in-water emulsions by loading the oil phase with water droplets [1, 3], they also show higher instability. Furthermore, plastic properties are desirable for DEs when they are used for replacing animal fat in food products, which is highly saturated and solid at room temperature, to mimic adipose tissue in its physical properties. However, DEs do not provide solid-like texture unless oil droplets are closely packed [4, 5]. Gelling of the internal [3, 6,7] or external aqueous phase [8–13], together with the structuring of the oil phase [3, 14] are among the different strategies attempted for stabilizing DEs [15]. However, the gelation of the external aqueous phase, embedding the emulsion droplets in a continuous hydrogel matrix, is the most promising strategy to also provide a fat analog with solid-like properties [4]. Different compounds such as pectin, alginate, gellan gum, glycyrrhizic acid nanofibrils have been incorporated into the external aqueous phase of DE to form double emulsion gels [4, 7, 16]. Cold-set gelation is an attractive alternative for gelling DEs when heat-labile bioactive compounds are encapsulated [17]. In this context, the incorporation of microbial transglutaminase and gelatin into W_2_ led to the formation of thermostable cold-set double emulsion gels [9, 10, 12, 17]. Transglutaminase covalently binds different protein residues, leading to the formation of relatively thermostable gels [4].

Betalains are natural water-soluble pigments composed of two structural groups: betacyanins (reddish-purple) and betaxanthins (orange–yellow). Although several betalains sources have been described (amaranth, djulis, some cactaceas such as cactus pear, pitayas, pitahayas; [18], red beet (*Beta vulgaris* L.) is the main source of commercial betalains, which is classified as additive E-162 (EU) and 73.40 (FDA, USA). However, red beet has an earth-like flavour attributed to geosmin and 3-*sec*-butyl-2 methoxypyrazine, and besides it has high nitrate levels [19]. In this context, the purple cactus pear *(Opuntia ficus-indica*) is an interesting alternative as a source of betalains, where betanin is the main colourant compound, and indicaxanthin and isobetanin have been detected at a low level [20]. Furthermore, betalains have been associated with several beneficial effects on human health, such as protection against oxidative stress-related and cardiovascular diseases [18]. The stability of betalains is an important aspect to consider for using these bioactive compounds in foods, since they are affected by pH, concentration of buffer, water activity, light, oxygen, temperature and enzymatic activities [21, 22]. Encapsulation technology could be a useful strategy to prevent betalains degradation in food matrices [23] (Khan, 2016). Among the different encapsulation technologies used for betalains encapsulation, spray-drying is one of the most commonly used [24]. However, the encapsulation of betalains in DEs, mostly from reed beet, is scarce [25–27] and, to the best of our knowledge, gelled DEs have not been evaluated as encapsulation strategy. Gelled DEs would allow widening the application of betalains as antioxidants and natural food colorants in food matrices where solid-like properties are desirable. The aim of this work was to study the stability of betanin from a purple cactus pear extract when they are encapsulated in the internal aqueous phase of DEs with a liquid or gelled external aqueous phase. Gelation of the external aqueous phase was addressed with gelatin (G) or a mixture of gelatin and transglutaminase (T).

## Materials and methods

### Materials

Purple cactus pear pulp (*Opuntia ficus-indica*) was obtained from Antumapu Experimental Station (University of Chile, Santiago, Chile). Olive oil (Team Foods S.A., Chile), linseed oil (Nutra Andes Ltda., Chile) and fish oil (Spess S.A., Chile) were used as lipid phase of DE Polyglycerol ester of polyricinoleic acid (PGPR; Dimerco S.A., Chile) and Tween 80 (Sigma-Aldrich, Chile) were used as lipophilic and hydrophilic emulsifiers, respectively. Bovine gelatin (220 Bloom, Blumos, Chile) and microbial transglutaminase (Activa GS, Ajinomoto, Japan; 47–82 units of hydroxamate/g) were used for gelling DE.

### Preparation of cactus pear extract

An ethanolic extract was prepared from the cactus pear pulp according to Sáenz et al. [20]. Briefly, the pulp was defrosted and macerated with ethanol:water (1:1 v/v) for 12 h, with ratio pulp:solvent 1:1 (w/w). The supernatant of the extraction was collected, and the sediment was re-extracted twice for 2 h. The supernatants of the three extractions were combined and concentrated in a rotary evaporator (R-100, Büchi, Switzerland) at 40 °C, giving a cactus pear extract (CP) with a betanin concentration of 0.5 mg/mL.

The quantification of betanin in the concentrated extract was performed spectrophotometrically (Orion Aquamate 8000, Thermo Scientific, USA) at 538 nm (corrected by absorption at 600 nm), using a betanin standard calibration curve (0.2–6.7 mg betanin/mL; R^2^ = 1).

The betanin standard was obtained from the cactus pear extract according to Forni et al. [28]. Briefly, silica (70–230 Mesh ASTM, Merck) was activated at 150 °C for 1 h, allowed to cool for 15 min, packed in a glass column and conditioned with McIlvaine buffer (citrate phosphate buffer, pH 6.5). The cactus pear extract (6 mL) was applied in the column, and the McIlvaine buffer was used as mobile phase. The purple fraction was collected, and the isolation of betanin was ascertained by HPLC, according to Fernández-López and Almela [29], using a Merck Hitachi L-6200 pump, a Waters 996 photodiode-array detector and a C18 column (5 μm, 4.6 i.d. x 250 mm, Symmetry, Waters). The quantification of betanin in the fraction was performed spectrophotometrically (Orion Aquamate 8000, Thermo Scientific, USA) according to Stintzing et al. [30] by using this equation:

Betanin(mg/L)=A×DF×MW×1000/ε×l (1)

Where A is the absorption value, calculated from the difference between absorption values at 538 nm and absorption values at 600 nm, DF is the dilution factor, and l is the path length of the cuvette (1 cm). MW and *ε* are the molecular weight and molar extinction coefficient of betanin, respectively (MW 550 g/mol; *ε* = 60 000 L/(mol cm) in H_2_O).

### Preparation of DEs

Double emulsions were formulated following a two-step emulsification process [31] with slight modifications. The cactus pear extract with a betanin concentration of 0.5 mg/mL was used as the internal aqueous phase (W_1_). A blend of olive, linseed and fish oils (70:20:10, w/w/w) was used as lipid phase [31]. PGPR was added to the oil blend (6%, w/w), and a W_1_/O coarse emulsion was prepared by drop-wise addition of W_1_ (20%) to the lipid phase (80%) using a blender (Thermomix, Vorwek, Germany; 3250 rpm, 5 min, 40 °C). This W_1_/O emulsion was homogenized twice at 55,000 kPa (first stage)/7,000 kPa (second stage) with a two-stage high pressure homogenizer (Panda Plus 2000, GEA Niro Soavi, Italy). The W_1_/O/W_2_ coarse emulsion was prepared by gradually addition of W_1_/O (40%) to W_2_ (60%) containing Tween 80 (2% w/w) at 700 rpm and room temperature. This W_1_/O/W_2_ coarse emulsion was homogenized twice at 15,000 kPa (first stage)/3,000 kPa (second stage) to obtain the W_1_/O/W_2_ fine emulsion, with 40 mg betanin/kg DE. This W_1_/O/W_2_ fine emulsion was divided in three batches. One batch was aliquoted in 50 mL tubes that were closed and chilled at 4 °C in darkness (DE-CP). This emulsion (DE-CP) maintained a liquid external aqueous phase. The external aqueous phase was gelled with gelatin in the second batch (DE-CP-G). Bovine gelatin (4% w/w) was dissolved in the W_2_ of the freshly prepared DE-CP at 40 °C for 10 min. In the case of the third batch, W_2_ was gelled with gelatin and transglutaminase (DE-CP-GT). Bovine gelatin (4% w/w) and microbial transglutaminase (2% w/w) were dissolved in the W_2_ of the freshly prepared DE-CP at 40 °C for 10 min [10]. DE-CP-G and DE-CP-GT were aliquoted in 50 mL tubes that were closed and chilled at 4 °C in darkness. After a 12 h overnight (day 0), the three emulsion systems (DE-CP, DE-CP-G and DE-CP-GT) were subjected to a thermal treatment (70 °C/30 min).

### Characterization of DEs

#### Determination of droplet size and size distribution of DE-CP

The size and distribution of oil droplets in DE-CP were determined using a Malvern Mastersizer 3000 particle size analyzer (Malvern Instrument Ltd., Worcestershire, UK), equipped with a He-Ne laser (λ = 633nm) and a measurement range 0.02–2000 μm. Particle size calculations were based on the Mie Scattering theory. Ten measurements were performed on each emulsion. The results were expressed as the average of the volume-weighted mean globule size (*D*_4,3_).

#### Confocal laser scanning microscopy

Morphology of freshly prepared DE with a liquid (DE-CP) or gelled (DE-CP-G and DE-CP-GT) external aqueous phase was evaluated by confocal laser scanning microscopy (CLSM, LSM 700, Carl Zeiss, Germany) at 40x magnifications. The oil phase of DEs was fluorescently labelled with Nile red (0.02% w/w). Samples were placed onto microscope slides, covered with a cover glass and subjected to 488 nm excitation wavelength, while fluorescent signal was collected at 580 nm. The software used for the CLSM imaging was ZEN 2012 (Blue Edition, Carl Zeiss, Germany).

#### Encapsulation efficiency of DE-CP

The EE of betanin was determined in DE-CP after preparation, by measuring the betanin concentration in W_2_ spectrophotometrically. A 3-fold dilution of DEs in water with the adjusted osmolarity was centrifuged (2500 g, 30 min) to obtain W_2_ that was filtered (0.22 μm, Millipore filter). Betanin was quantified as described for the concentrated extract, using a betanin standard calibration curve (0.2–6.7 mg betanin/mL; R^2^ = 1). The EE was defined as the percentage of betanin in W_1_ that remained in the primary emulsion W_1_/O after the second emulsification process, and it was calculated following the equation (Eq 2):

$$\text{EE}=100-\left({\text{Betanin}}_{\text{W}2}\;\text{x}\;100\right)/{\text{Betanin}}_{0\text{T}}$$ (2)

Where Betanin_W2_ is the betanin concentration recovered in W_2_ after preparation, Betanin_0T_ is the total concentration of betanin in the system after preparation, determined as described in the next section.

The encapsulation stability (ES) of betanin was also determined at specific time intervals during storage at 4 °C. ES was defined as the percentage of betanin in W_1_ during the storage, and it was calculated according to Eq 3:

$$\text{ES}=100-\left({\text{Betanin}}_{\text{W}2(\text{t})}\;\text{x}\;100\right)/{\text{Betanin}}_{\text{t}}$$ (3)

Where Betanin_W2(t)_ is the betanin concentration recovered in W_2_ at specific storage time (t), and Betanin_t_ is the total concentration of betanin in the system at specific storage time (t), determined as described in the next section.

#### Total content of betanin in DEs

The total content of betanin was determined in DEs with a liquid (DE-CP) or gelled (DE-CP-G and DE-CP-GT) W_2_ at specific time intervals during storage time at 4 °C, and after the thermal treatment (70 °C/30 min) at day 0. For that, 2 g of each system were homogenized sequentially with methanol (2.8 mL), chloroform (2.8 mL) and distilled water (1.4 mL) by using a rotor-stator (T18 IKA, Germany) at 20,000 rpm for 15 s (each solvent). Afterwards, the samples were centrifuged (9,610 g for 30 min) at 4 °C. The supernatants were collected, and the total content of betanin was determined spectrophotometrically as described for the concentrated extract, using a betanin standard calibration curve (0.2–6.7 mg betanin/mL; R^2^ = 1).

#### Rheology of DEs

Dynamic rheological analyses were performed on the three systems (DE-CP, DE-CP-G and DE-CP-GT), using a rheometer (HR-1 Discovery Hybrid Rheometer, TA Instruments, USA), equipped with a cone-plate geometry (1.008°, 60 mm diameter, 27 μm gap for DE-CP) and a parallel-plate geometry (40 mm diameter, 500 μm gap for DE-CP-G and DE-CP-GT). Samples were analyzed after cold maturation at 5 °C for 18 h, and they were allowed to stand for 5 min before rheological analysis. Strain amplitude sweeps (from 0.01 to 10%) at a frequency of 1 Hz were performed to determine the linear viscoelastic (LVE) region. The strain amplitude was set at γ = 0.8%, within the LVE, in the three systems for both frequency and temperature sweeps. For frequency sweeps, the samples were subjected to stress that varied harmonically with time, at variable frequencies from 0.1 to 10 Hz and 20 °C. The viscous modulus (G”; Pa) in the case of DE-CP, and both the elastic modulus (G’; Pa) and G” in the case of DE-CP-G and DE-CP-GT, were plotted as a function of frequency. Furthermore, temperature sweeps were run in DE-CP-G and DE-CP-GT from 5 °C to 50 °C (2 °C/min), at a frequency of 1 Hz. G’, G” and phase angle (δ) values were plotted as a function of temperature. Results were average of at least three measurements.

#### Degradation rate constants of betanin in DEs during storage

The aliquoted samples (DE-CP, DE-CP-G and DE-CP-GT) were stored at 4 °C in absence of light for 28 days (DE-CP-GT) and 49 days (DE-CP and DE-CP-G). Samples were withdrawn at specific time intervals (in triplicate), and the total betanin content was determined as described above (Total content of betanin in DEs). Data were fitted by a first-order kinetic model, ln C = ln C_0_-k_(t)_. Degradation rate constants (k_*obs*_) were obtained from the slope of a plot of the natural log of the percentage retention of betanin *vs*. time.

#### Color and pH of DEs

Color measurements were performed with a portable spectrophotometer (MiniScan XE Plus, HunterLab, USA) on DE-CP, DE-CP-G and DE-CP-GT at specific time intervals during storage at 4 °C, and after the thermal treatment (70 °C/30 min) at day 0. Hue angle [h° = (arctan b*/a*)] was calculated from a* and b* values using illuminant D_65_.

To determine pH, the samples (10 g) were homogenized with 90 mL of distilled water and pH values were measured by using a pH metro (HI 5221, Hanna Instruments, USA).

#### Identification of betanin degradation products in DEs

The samples were analyzed according to Herbach et al. [32] with some modifications, at the end of the storage period at 4 °C and after the thermal treatment (70 °C/30 min) at day 0. An Ultra-High Performance Liquid Chromatography (UPLC) Ultimate 3000 RSLC system was used, coupled to a Linear Ion Trap Mass Spectrometer LTQ XL (Thermo Scientific, USA), with a C18 column (1.7 μm particle size, 3 mm i.d. x 100 mm; Kinetex, Phenomenex, USA). The mobile phase A was 0.1% formic acid in MiliQ water, and 0.1% formic acid in acetonitrile was the mobile phase B. Separation was performed at a flow rate of 0.3 mL/min, using a gradient program as follows: 5% B (1 min), 5% B to 80% B (19 min), 80% B (7 min), 80% B to 5% B (5 min). The mass detection was carried out using electrospray ionization (ESI), with the spray voltage set at 3 kV (at 300°C). Detection was performed in full scan mode in the 100–1000 m/z range in positive mode. MS2 spectra were obtained using He for collision-induced fragmentation (CID) with a normalized collision energy of 35 units and detection of fragments in full scan mode.

### Statistical analysis

All the experiments were performed in triplicate. Analysis of variance (ANOVA) and Tukey’s multiple range tests were applied to determine statistical differences among samples, using Statgraphics Centurion XVI software (Statistical Graphics Corporation, USA).

## Results and discussion

### Size and size distribution of DE-CP

**Fig 1:**
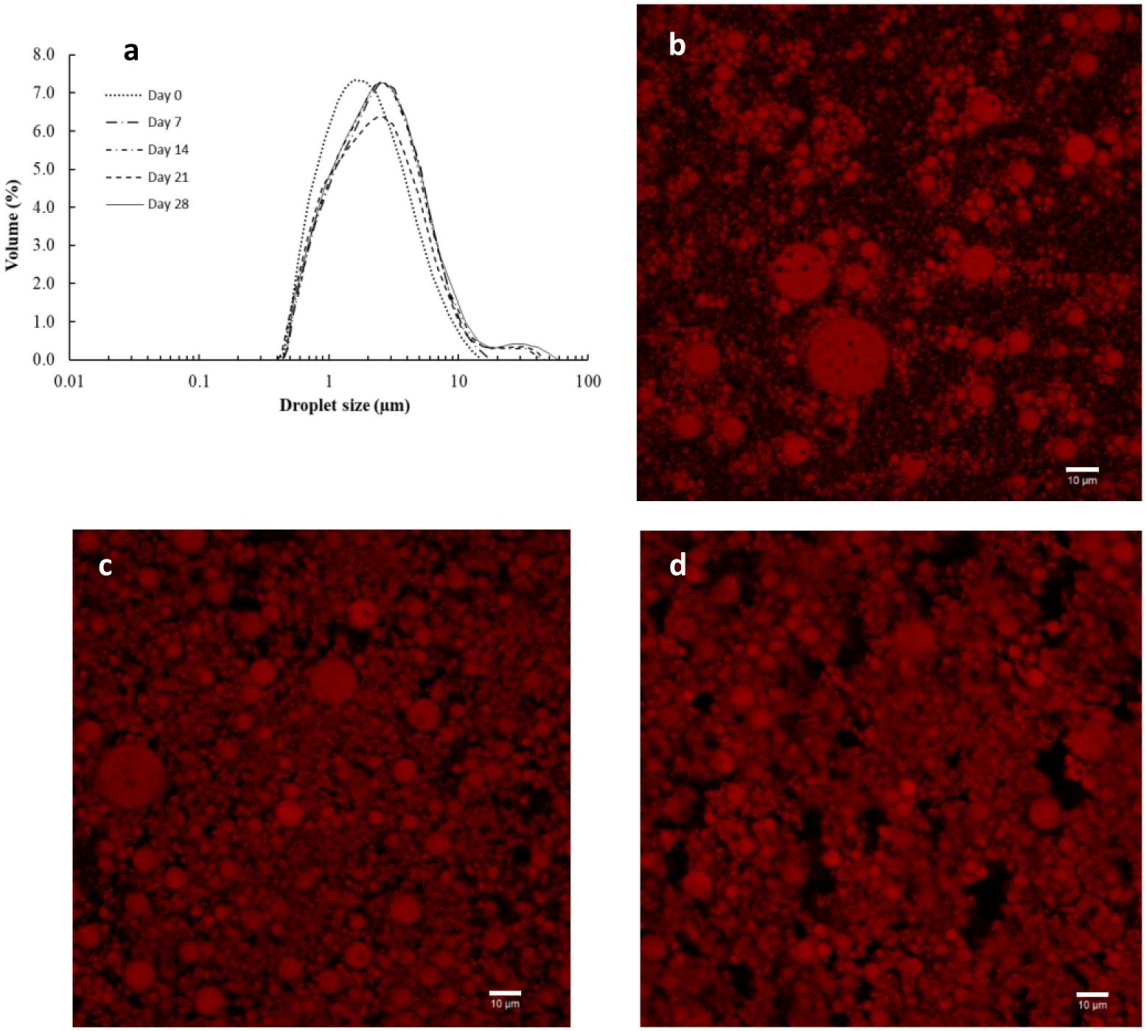
Size distribution and microstructure of DEs. Size distribution of DE-CP during storage at 4 °C (1a): Day 0 (····), day 7 (- -), day 14 (-·-·-), day 21 (- - -), day 28 (—). Microstructure of DE-CP (1b), DE-CP-G (1c) and DE-CP-GT (1d) after their preparation.

The size distribution and *D*_4,3_ values of DE-CP are shown in Fig 1a and Table 1, respectively.

**Table 1:** Droplet size (*D*_4,3_) of DE-CP during storage at 4 °C, and after a thermal treatment (70 °C/30 min) applied periodically during storage at 4 °C.

	*D*_4,3_	
Days	4°C	After 70°C/30 min
**0**	2.56 ± 0.21^a,x^	2.37 ± 0.05^x^
**7**	3.00 ± 0.04^b,y^	1.94 ± 0.01^x^
**14**	3.32 ± 0.07^c,y^	2.16 ± 0.16^x^
**21**	3.34 ± 0.05^c,y^	1.51 ± 0.03^x^
**28**	3.83 ± 0.12^d,y^	3.16 ± 0.10^x^

Different letters (a-d) indicate significant differences among days of storage at 4 °C. Different letters (x-y) indicate significant differences between treatments for the same day of storage.

DE-CP showed a monomodal size distribution at day 0, with oil globule populations in the range 0.4–15.1 μm, and *D*_4,3_ values of 2.56 μm. Larger oil droplets were reported for DEs with betanin (purified from red beet) encapsulated in W_1_ (*D*_3,2_ 45.8 μm; [27]) or DEs with a spray-dried beetroot juice encapsulated in W_1_ (*D*_3,2_ 72.4 μm; [27]), both obtained by microchannel emulsification. In other study, where DEs with a beetroot juice encapsulated in W_1_ were prepared by hybrid membrane premix emulsification system, a droplet size (*D*_3,2_) of 20.4 μm was obtained [26]. Thus, several factors may affect both droplet size and size distribution in DEs, such as oil composition and viscosity of the phases, type and concentration of emulsifiers, processing conditions (homogenization), type and source of bioactive compounds encapsulated in W_1_ [27, 33, 34].

Storage of DE-CP at 4 °C produced some changes both in oil globule size and oil globule size distribution. Larger oil globules, with sizes in the range 0.4–58.7 μm, were found at day 28 of storage. The shift towards larger sizes, may be due to coalescence of the oil globules during storage. The increase in size throughout the storage led to a slight but significant increase of *D*_4,3_ values (p≤0.05) from 2.56 (day 0) to 3.83 (day 28) (Table 1), since *D*_4,3_ is sensitive to the presence of large particles within a polydisperse system [34]. At the end of the storage, approximately 95% of the oil globule population had sizes below 10 μm, denoting high emulsion stability during storage.

As DE-CP is intended to be used as food ingredient, it was subjected to 70 °C for 30 min, to mimic a conventional thermal treatment used in the food industry. The heating (70 °C for 30 min) of DE-CP at day 0 did not involve noticeable changes in *D*_4,3_ values (p>0.05; Table 1), denoting an excellent thermal stability. However, the heating of DEs from 7 days of storage at 4 °C onwards, led to the formation of small oil globule populations with smaller sizes and, therefore, to a slight but significant decrease (p≤0.05) of *D*_4,3_ values (Table 1). This reduction in size may be related to the breakup of the oil globules and the appearance of new smaller oil globules [27], with the consequent partial loss of W_1_ and release of the cactus pear extract. These results agree with other studies [35, 36], where DEs showed a decrease of thermal stability with storage time at 4 °C. Pagano et al. [27] also found a reduction in size of DEs with betanin encapsulated in W_1_ when they were continuously stored at 25 °C and 60 °C for 7 days.

### Microstructure of DEs

Fig 1b–1d shows the microstructure of DEs with a liquid (DE-CP, Fig 1b) or gelled external aqueous phase (DE-CP-G, Fig 1c; DE-CP-GT, Fig 1d). The three systems showed the characteristic microscopic structure of DE, with oil globules containing smaller water droplets inside. Therefore, the gelation process did not affect the typical multi-compartmentalized structure of DE. However, the incorporation of both gelatin (Fig 1c) and gelatin/transglutaminase (Fig 1d) to the external aqueous phase of DE led to a higher packing of oil globules. Flaizt et al. [12] also reported that the incorporation of gelatin and transglutaminase to the continuous phase of DEs with hydroxytyrosol encapsulated in W_1_ did not affect the typical DE compartmentalized structure, but oil globules were closely packed.

### Rheology of DEs

**Fig 2:**
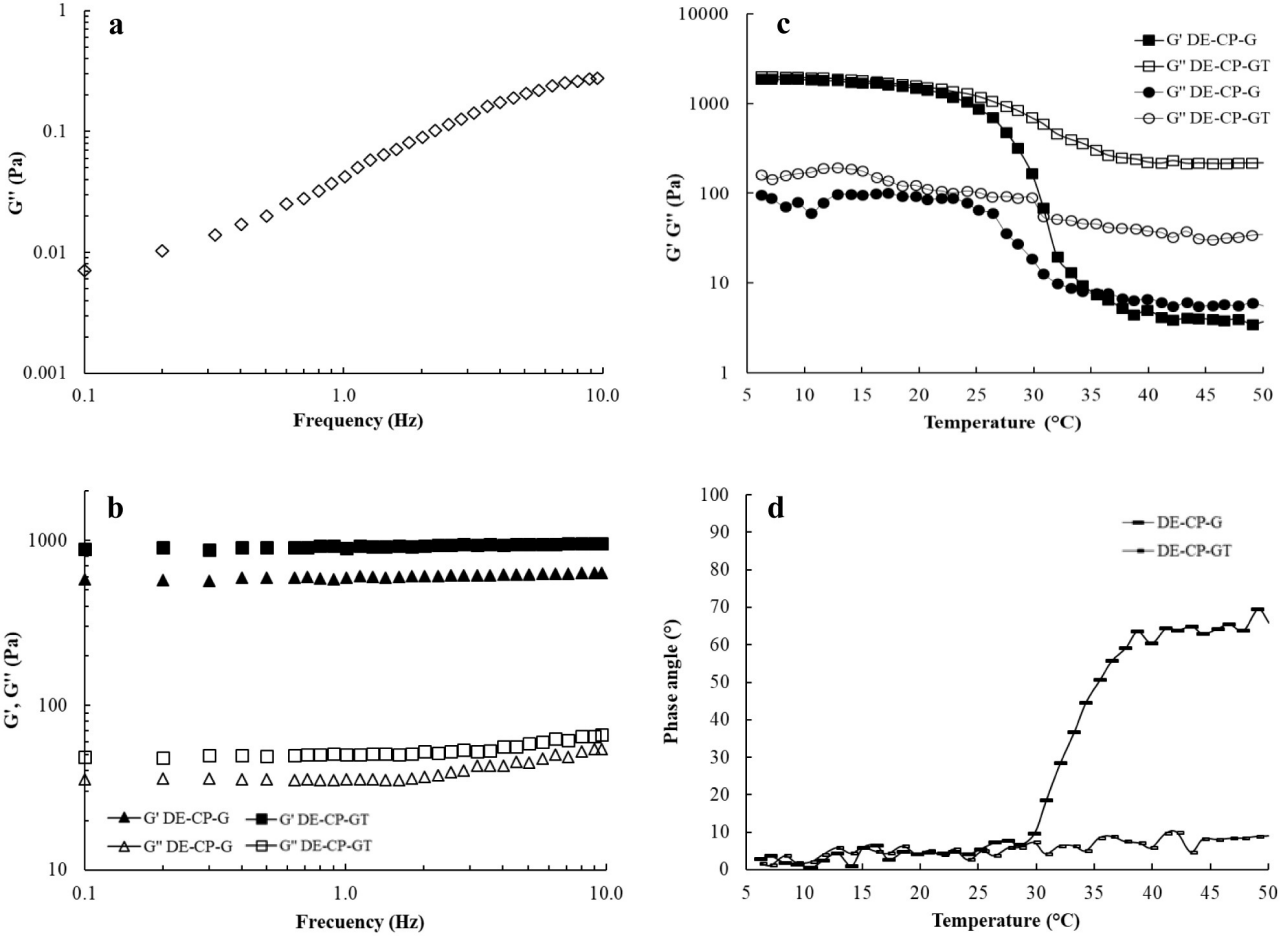
Rheology of DEs. Frequency sweep run in DE-CP (G”, ◊; 2a), DE-CP-G (G’, ▲; G”, Δ; 2b) and DE-CP-GT (G’, ■; G”, □; 2b). Evolution of G’ (2c) and δ (2d) values during temperature sweep in cold-maturated DE-CP-G (G’, ▲; δ, —) and DE-CP-GT (G’, ■; δ, -).

Dynamic oscillatory shear tests were performed on DEs to characterize its viscoelastic properties. The dynamic mechanical spectra of DE-CP showed that G” values were higher than G’ values all over the frequency range. Furthermore, G’ values were very low and irregular and only G” values were plotted as a function of frequency (Fig 2a).

Therefore, DE-CP showed a viscous behavior within the frequency range studied, and G” showed high frequency dependence, in agreement with the results reported for DE in other studies [36, 37]. A dominant viscous behavior was also reported in DEs formulated at the same volumetric ratio of W_1_/O in W_2_ (40/60), with chia, sunflower and olive oils, attributed to the high water content in DE [36, 37]. According to Russ-Murphy [38], high G” to G’ ratios are typically found in dilute solutions, with both moduli showing a strong frequency dependence. In contrast, a dominant elastic behavior has been reported for DEs formulated at higher volumetric ratios of W_1_/O in W_2_ (70/30 and 80/20; [5]. The behavior of DE-CP-G and DE-CP-GT was predominantly more elastic than viscous, since G’ values were higher than G” values all over frequency range (Fig 2b). Furthermore, both G’ and G” showed low frequency dependence, contrary to DE-CP, since the influence of frequency is reduced by increasing the structural strength of these systems [9]. According to the low values obtained for δ in both DE-CP-G and DE-CP-GT throughout the frequency range (δ ≤ 5°), both samples can be considered gels with a strong structure at 20 °C.

The viscoelastic properties of the gelled DEs were also analyzed after cold maturation at 5 °C for 18 h. Fig 2 shows the values obtained for G’ and G” (Fig 2c) and δ (Fig 2d), during heating from 5 to 50 °C. DE-CP-G showed a melting temperature (G” > G’) of 35.5 °C (Fig 2c), and high δ values at 50 °C (δ ~ 66°; Fig 2d). Under subsequent cooling from 50 °C to 5 °C, DE-CP-G showed a good refolding ability, reaching high G’ values (G’ = 1349 Pa) and low δ values (δ = 1.6°) at 5 °C, denoting the thermoreversible property of this gelled DE (S1 Fig). As expected, the incorporation of gelatin and transglutaminase in the W_2_ of DE led to the formation of a thermostable gel, as can be deduced from δ values found in DE-CP-GT throughout the heating range (δ < 10°; Fig 2d). However, a ten-fold decrease in G’ values was found during heating, probably due to the melting of the gelatin fraction not involved in the thermostable gel. In contrast, Freire et al. [9] found a slight decrease in G’ values in thermostable DEs, gelled with gelatin (4% w/w) and transglutaminase (2% w/w), when they were heated from 20 °C to 80 °C; although these samples were analyzed after 30 days chilling, much longer time for enzyme activity.

### Stability of betanin in DEs

Table 2 shows the EE, ES and evolution of total betanin retention in DE-CP, DE-CP-G and DE-CP-GT during storage at 4 °C, as well as betanin degradation rate constants for the emulsion systems studied. Encapsulation efficiency of betanin in DE-CP was 87.7% at day 0.

**Table 2:** Encapsulation efficiency (EE), encapsulation stability (ES) and evolution of betanin total retention in DE-CP, DE-CP-G, DE-CP-GT during storage at 4 °C.

Days	DE-CP				DE-CP-G	DE-CP-GT
	EE (%) (day 0) ES (%) (days 7–49)	Betanin retention (%)				
		W_1_	W_2_	Total	Betanin retention (%)	Betanin retention (%)
0	87.7±0.1^a^	100^a^	100^a^	100^a^	100^a^	100^a^
7	86.5±0.6^bc^	90.7±1.1^ab^	100.7±3.1^a^	91.9±0.6^ab^	91.6±0.9^ab^	40.48±1.33^b^
14	86.2±0.3^cd^	82.3±3.6^bc^	94.2±0.7^b^	83.7±3.2^bc^	89.2±2.4^bc^	19.72±0.07^c^
21	85.3±0.7^cde^	74.1±5.2^cd^	91.4±0.1^bc^	76.2±4.6^cd^	80.4±0.1^cd^	14.23±0.30^d^
28	84.6±0.1^de^	67.1±0.6^de^	87.0±0.1^cd^	69.6±0.5^de^	79.6±5.2^d^	6.80±0.44^e^
35	83.9±0.1^e^	59.4±0.7^e^	84.2±0.3^d^	62.4±0.6^ef^	69.4±1.6^e^	
49	80.9±0.8^f^	44.5±0.1^f^	78.0±0.7^e^	48.6±0.0^g^	59.0±1.4^f^	
k_*obs*_ (days ^-1^)				14.6±0.1x10^-3a^	11.0±0.2x10^-3a^	90.2±4.2x10^-3b^

W_1_: internal aqueous phase; W_2_: external aqueous phase; DE-CP: double emulsion with a liquid external aqueous phase and a cactus pear extract encapsulated in W_1_; DE-CP-G: double emulsion with a gelled external aqueous phase (gelatin) and a cactus pear extract encapsulated in W_1_; DE-CP-GT: double emulsion with a gelled external aqueous phase (gelatin and transglutaminase) and a with cactus pear extract encapsulated in W_1_.

Different letters (a-f) in the same column indicate significant differences among days of storage. Different letters (a-b) in the row k_*obs*_ indicate significant differences among samples.

Slightly higher encapsulation efficiency values (89.1%) were reported for DEs loaded with red beet extract and obtained with a high-speed mixer [25]. Eisinaite et al. [26] also reported higher EE than this study in food-grade DEs containing native (98.62%) and concentrate red beet juice (100%). Several factors could influence the EE in DEs, such as the nature of the bioactive compound (molecular weight, water solubility, polarity), the composition of DE and the emulsification method used [7, 12]. The ES of betanin in DE-CP showed a slight decrease during the storage, from 87.7 to 80.9% (p≤0.05). However, the ES values did not explain the internal changes of betanin content in DE-CP, since the content of betanin in both W_2_ and W_1_ showed a marked decrease during the storage (Table 2), due to the release and/or degradation of betanin from W_1_, and degradation of betanin in W_2_.

The degradation of total betanin followed first order reaction kinetics at 4 °C in all the studied systems. The same kinetic order was reported for the degradation of betanin in water solution (pH = 4) at 85 °C [39], degradation of betacyanins from *Amaranthus* at temperatures in the range 40–100 °C [40] and betanin from beet root in cow milk at 70, 80 and 90 °C [41]. Betanin degradation rate constants (k_*obs*_) were obtained from the slope of a plot of the natural log of the betanin retention (%) *vs*. time (days), with coefficients of determination (R^2^) above 0.98 (S2 Fig). The betanin degradation was significantly higher in DE-CP-GT (90.2 x 10^−3^ days^-1^) than in DE-CP (14.6 x 10^−3^ days^-1^) and DE-CP-G (11.0 x 10^−3^ days^-1^) (Table 2). The differences in degradation rate constants among systems may be related to their pH values. At day 0, DE-CP and DE-CP-G showed pH values of 6.42 ± 0.07 and 6.35 ± 0.02, respectively; whereas DE-CP-GT showed pH values of 7.54 ± 0.12, due to the incorporation of the transglutaminase preparation. Similar pH values were reported for DEs gelled with transglutaminase, and hydroxytyrosol encapsulated in W_1_ (7.41 ± 0.17, [9]). Along the storage time at 4 °C, an increase of pH values was observed in all the systems, reaching values of 6.82 ± 0.02 and 6.69 ± 0.03 in DE-CP and DE-CP-G, respectively, at day 49 of storage. The pH values increased faster in DE-CP-GT, reaching values of 7.98 ± 0.03 at day 28 of storage, which may be related to the enzyme activity, since it catalyzes the attachment of primary amines to protein- or peptide-bound γ-carboxamides under ammonia release [42]. Therefore, though DE-CP-GT showed higher thermal stability according to its rheological behavior (Fig 2c and 2d), it was not suitable for betanin retention.

As mentioned above, these systems (DE-CP, DE-CP-G and DE-CP-GT) were subjected to thermal treatment (70 °C/30 min) after 12 h overnight (day 0) to mimic a conventional thermal treatment used in the food industry. A similar loss of betanin was found in DE-CP and DE-CP-G, reaching values of 40.6 ± 3.4% and 38.1 ± 5.6%, respectively. However, DE-CP-GT showed the highest betanin degradation, with 55.7 ± 3.2%. The higher betanin degradation in DE-CP-GT may be related to its pH values, since thermal stability of betanin is decreased at pH values outside the range 3–7 [21]. Several factors have been reported to affect the chemical stability of betanin, such as pH, water activity, oxygen, light, metals, antioxidant and temperature [21, 22]. In this study, two isomers of betalamic acid (bright yellow, [M+H]^+^ = 212) and *cyclo*-Dopa 5-*O*-β-glucoside (colorless, [M+H]^+^ = 358), 17-decarboxy-betanin (orange-red, [M + H] + = 507) and neobetanin (orange-red, [M + H] + = 549) were identified as betanin degradation products by UHPLC-MS/MS in the three samples at the end of the storage time at 4 °C and in the corresponding samples subjected to thermal treatment (70 °C for 30 min) (Table 3).

**Table 3:** Degradation products of betanin identified in DE-CP, DE-CP-G and DE-CP-GT at the end of storage at 4 °C, and in the samples subjected to thermal treatment (70 °C/30 min).

Compound	RT (min)	m/z [M+H]^+^	UHPLC-MS-MS^2^ experiment m/z (% base peak)
Betanidin 5-*O*-β-glucoside (Betanin)	6.02	551	MS^2^ [551]: 389 (100)
17-Decarboxy-betanin	6.13	507	MS^2^ [507]: 345 (100)
(iso)-Betalamic acid	6.53	212	MS^2^ [212]: 194 (12), 166 (100)
Isobetanidin 5-*O*-β-glucoside (Isobetanin)	6.78	551	MS^2^ [551]: 389 (100)
Neobetanidin 5-*O*-β-glucoside (Neobetanin)	7.55	549	MS^2^ [549]: 387(100)
*cyclo*-Dopa 5-*O*-β-glucoside	10.33	358	MS^2^ [358]: 341 (100)

RT: Retention time

The degradation of betanin through hydrolysis of aldimine bond, giving *cyclo*-Dopa 5-*O*-β-glucoside and betalamic acid, has been proposed as the main pathway for decomposition and this may be favored in systems with high water activity such as W_1_/O/W_2_ double emulsions [21, 22, 24]. Alkaline conditions, such as those in DE-CP-GT, have been also reported to bring out aldimine bond hydrolysis of betanin [21]. In other study, the same degradation products of betanin were identified at both pH 2 and pH 9, with a difference in their formation rate [22]. Furthermore, betalamic acid, *cyclo*-Dopa 5-*O*-β-glucoside and neobetanin have been identified as thermal degradation products of betanin in juices from purple pitaya (*Hulocereus polyrhizus*) or red beet [32, 43].

### Color stability of DEs

**Fig 3:**
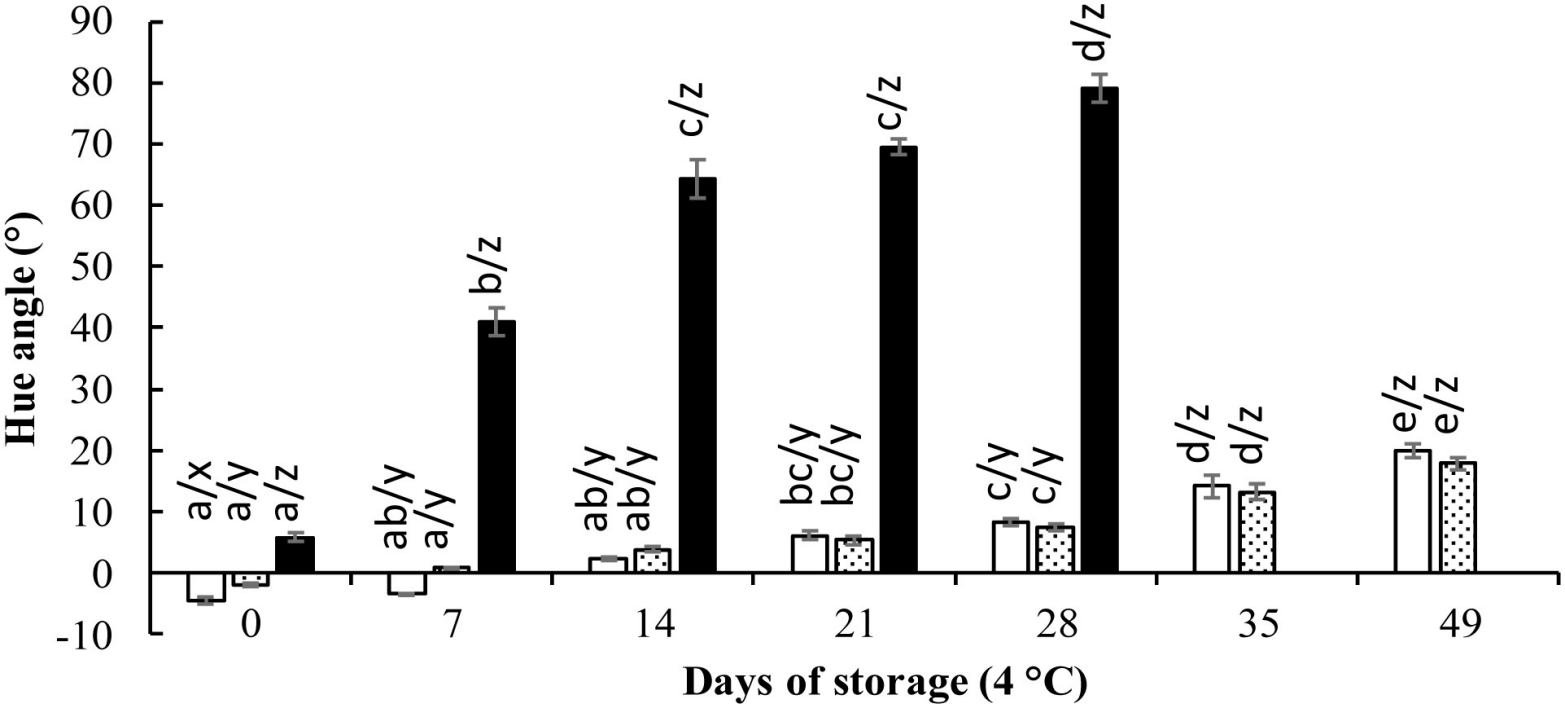
Evolution of hue angle (°) in DEs during storage at 4 °C. DE-CP (white bar), DE-CP-G (dotted bar), DE-CP-GT (black bar). Different letters (x-z) indicate significant differences (p≤0.05) among samples at the same day of storage. Different letters (a-e) indicate significant differences (p≤0.05) throughout the storage time for the same sample.

Fig 3 shows the evolution of hue angle values for DE-CP, DE-CP-G and DE-CP-GT during storage at 4 °C. DE-CP-GT showed the highest hue values at day 0, followed by DE-CP-G and DE-CP.

Starting from red-purple tonality, with hue angle values in the range 4.58–5.83°, increasing hue angle values were observed with storage time (p≤0.05), reflecting a color shift towards yellow in all the systems, especially in the case of DE-CP-GT that reached hue angle values of 79° at day 28 of storage, in agreement with the yellow and orange-red degradation products of betanin identified by UPLC-MS (Table 3). A similar evolution of hue angle values was found in DE-CP and DE-CP-G, reaching values around 20° (p>0.05) at day 49 of storage. A high correlation between hue angle values and betanin retention was found in all the systems (R^2^ = 0.98 for DE-CP and DE-CP-G; R^2^ = 0.97 for DE-CP-GT; S3 Fig), suggesting that, under these experimental conditions, betanin retention could be predicted from hue angle values. In other study, a high correlation was reported between betalains content from beetroot and both L* and chroma values in colored milk during thermal treatment [41].

When the systems were subjected to thermal treatment (70 °C/30 min) after a 12 h overnight (day 0), a significant increase (p≤0.05) in hue angle values was observed in all the systems, indicating a shift towards the yellow region. A similar increase in hue angle values was found in DE-CP and DE-CP-G, reaching values of 14.5 ± 1.7° and 16.5 ± 0.8°, respectively; whereas DE-CP-GT reached values of 43.3 ± 2.4°. This increase in hue angle values was in agreement with the betanin degradation found due to thermal treatment, higher in the case of DE-CP-GT (55.7 ± 3.2%). Pagano et al. [27] reported that betanin from different sources, encapsulated in W/O/W emulsions, was also temperature sensitive, and a decrease of a* values together with an increase of b* values were reported with increasing temperature from 4 °C to 60 °C. These authors suggested that the shift towards yellow colors was probably due to the formation of betalamic acid, decarboxylated derivatives or oxidation products [22], but an identification of degradation products of betanin was not carried out. In our study, yellow (betalamic acid and neobetanidin 5-*O*-β-glucoside) and orange-red (17-decarboxy betanin) degradation products of betanin were identified in the samples subjected to 70 °C for 30 min (Table 3), explaining the shift towards yellow colors. These compounds were also reported as degradation products of betanin in red beet juice subjected to thermal treatment at 85 °C, explaining the color shift towards yellow observed in the juice [32]. In contrast, Eisinaite et al. [26] reported that the red color (evaluated by a*) of DE with beet root juice as W_1_, was stable after thermal treatment at 70 °C and 75 °C, although these treatments were applied only for 5 min; while significantly decreased after thermal treatment at 85 °C and 90 °C for 5 min.

## Conclusions

The incorporation of gelatin or a mixture of gelatin and transglutaminase in the external aqueous phase of a DE, with a purple cactus pear extract encapsulated in the internal aqueous phase as colorant agent, led to the formation of cold-set gelled DEs with solid-like behavior, which is desirable for certain food applications of DEs such as replacement of animal fat, without affecting the typical multi-compartmentalized structure of DE. Betanin from cactus pear gave red-purple color to DEs with a liquid or gelled external aqueous phase. However, the gelling agent influenced the stability of betanin and color. In the case of DE-CP-GT, higher pH values favored the degradation of betanin during cold storage and decreased their thermal stability, leading to a shift towards yellow colors. Betalamic acid, *cyclo*-Dopa 5-*O*-β-glucoside, 17-decarboxy-betanin and neobetanin were identified by UHPLC-MS/MS as degradation products of betanin. DEs with an external aqueous phase gelled with gelatin protected betanin of a purple cactus pear extract from degradation, and, given their solid-like properties, were promising to be used as potential fat replacers in food products consumed at temperatures under the gelatin melting point.

## Supporting information

S1 Fig Refolding ability of DE-CP-G. (DOCX)

S2 Fig Betanin retention during storage at 4 °C. DE-CP (○), DE-CP-G (□) and DE-CP-GT (Δ) (n = 3).
(DOCX)

S3 Fig Correlation between betanin retention (%) and hue angle (°) in DE-CP, DE-CP-G and DE-CP-GT. (DOCX)
