# Supplementary figures and images for: Influence of gelation on the retention of purple cactus pear extract in microencapsulated double emulsions
Source: PLoS One. 2020 Jan 16;15(1):e0227866. doi: 10.1371/journal.pone.0227866 (PMC6964817; doi:10.1371/journal.pone.0227866)

**S1. Fig. Refolding ability of DE-CP-G**


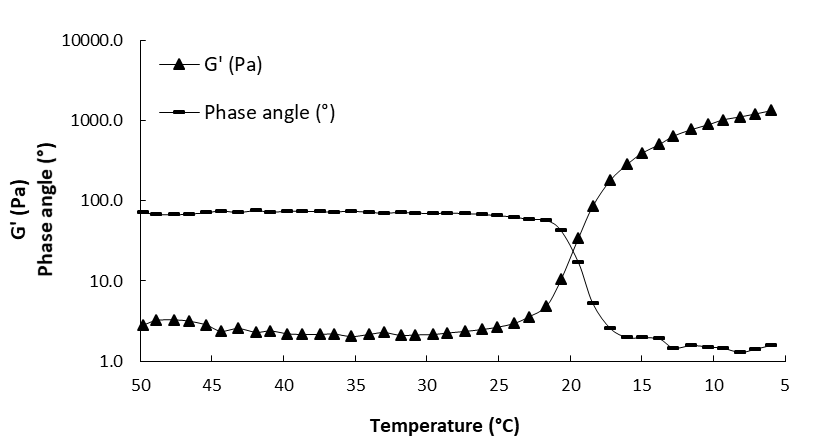

Supplement: S1 Fig — (DOCX) [file pone.0227866.s001.docx]

**S2 Fig.** Betanin retention in DE-CP (○), DE-CP-G (□) and DE-CP-GT ( ) during storage at 4 °C (n=3).


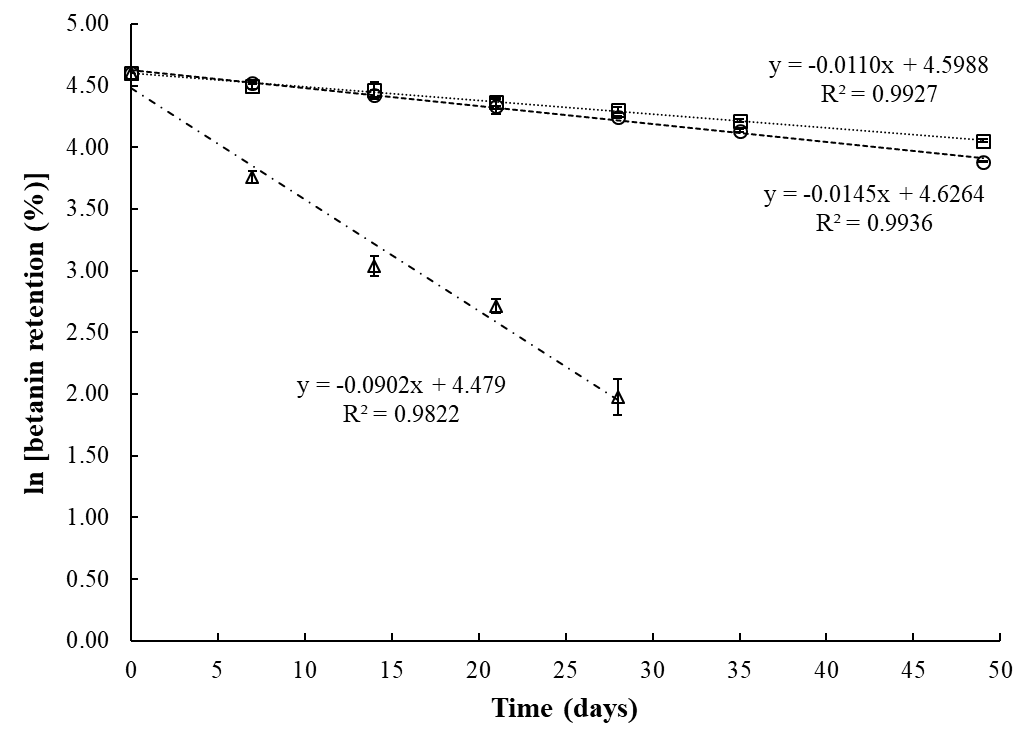

Supplement: S2 Fig — DE-CP (○), DE-CP-G (□) and DE-CP-GT (Δ) (n = 3). (DOCX) [file pone.0227866.s002.docx]

**S3 Fig.** Correlation between betanin retention (%) and hue angle (°) in DE-CP, DE-CP-G and DE-CP-GT.


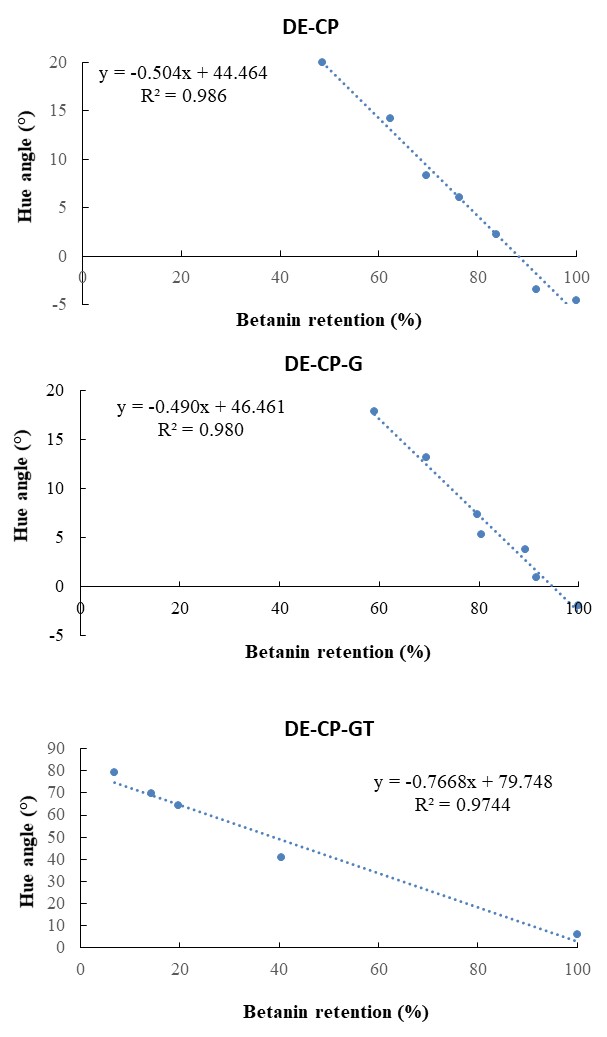

Supplement: S3 Fig — (DOCX) [file pone.0227866.s003.docx]
